# Advancing precision antibody-drug conjugate therapy: unique proteogenomic profiles of tumor subsets in non-small cell lung cancer

**DOI:** 10.1186/s40164-025-00685-w

**Published:** 2025-07-11

**Authors:** Edwin Lin, Ying-Chun Lo, Vivek Subbiah, Rajat Thawani, Aakash Desai

**Affiliations:** 1 Mayo Clinic, Rochester, MN USA; 2 Sarah Cannon Research Institute, Nashville, TN USA; 3 University of Chicago, Chicago, IL USA; 4 University of Alabama Birmingham, 1802 6th Ave South, Birmingham, AL 35294 USA

**Keywords:** Non-small cell lung cancer, Antibody-drug conjugates, Genomics, Transcriptomics, Proteomics, Precision medicine, Biomarkers

## Abstract

Antibody-drug conjugates (ADCs) represent a promising therapeutic strategy for non-small cell lung cancer (NSCLC), targeting tumor-specific antigens with precision. However, the molecular heterogeneity of NSCLC necessitates multiplex biomarker approaches to optimize ADC efficacy. This study utilized transcriptomics and proteomics to characterize NSCLC subtypes with distinct ADC target expression profiles. RNA-seq data from two independent cohorts (537 tumors, 59 controls; 338 tumors, 311 controls) identified clusters defined by overexpression of CEACAM5, MET, and TACSTD2, while normal lung tissue exhibited moderate TACSTD2 and FOLR1 expression. Chi-squared residual analysis revealed no significant associations with disease stage or driver mutations. Proteomic and transcriptomic data from 110 tumors and 101 controls demonstrated strong concordance. These findings highlight the potential of ADCs to target NSCLC subsets with distinct proteogenomic profiles, independent of disease stage or mutational status, underscoring their broad applicability in precision oncology.

**Supplementary Information:**

The online version contains supplementary material available at 10.1186/s40164-025-00685-w.

To the Editor.

Antibody-drug conjugates (ADCs) are sophisticated targeted therapies in the field of oncology, specifically designed to enhance the delivery of cytotoxic medication directly to tumor cells while minimizing systemic exposure. Essentially, ADCs are formed by linking monoclonal antibodies to potent cytotoxic drugs, allowing for the targeted eradication of cancerous cells [1]. A critical aspect of this progress has been integrating biomarker-based strategies, which have facilitated patient selection and enhanced the precision and effectiveness of ADCs. This study sheds light on the development of ADCs, particularly for treating tumors, focusing on non-small cell lung cancer (NSCLC) [2–4]. We propose that NSCLC has distinct subtypes defined by varying levels of expression of ADC targets. The objective of this study is to leverage these subclassifications to facilitate a precision-based approach to the selection of appropriate ADCs.

Our analysis of the transcriptomic profiles of ADC target expressions profiles from The Cancer Genome Atlas (TCGA) alongside the proteomic profiles of the antigens serving ADC targets from the Clinical Proteomic Tumor Analysis Consortium (CPTAC) has revealed unique subsets of tumors based on the expression patterns of the ADC targets under investigation. As we better understand these subtypes, we can lay the groundwork for personalized ADC therapies that have the potential to significantly enhance cancer treatment paradigms. This work is a beacon of hope for the future of NSCLC treatment, offering a more optimistic outlook for patients and healthcare professionals alike.

Our methodology was meticulous and comprehensive, involving a systematic search through multiple databases to pinpoint the ADC targets specifically within the domain of NSCLC. We evaluated RNA-sequencing data from The Cancer Genome Atlas (TCGA) to assess gene expression levels, encompassing 537 lung adenocarcinoma (LUAD) samples alongside 59 standard tissue samples [5]. The findings were subsequently validated using an independent cohort from the Clinical Proteomic Tumor Analysis Consortium (CPTAC) pertaining to lung adenocarcinoma [6]. Bioinformatics pipelines were extensively used for RNA-sequencing and somatic variant analysis, which involved utilizing tools like DESeq2 for conducting differential expression assessments. This meticulous approach instills confidence in the robustness of our findings.

From 35 scrutinized studies, we extracted relevant data concerning the target antigens associated with various ADCs (Supplementary Table 1). Notably, the ADC targets we identified included TROP2, MET, CEACAM5, HER2, HER3, Nectin4, PTK7, FRα, and B7H3. We revealed significant disparities in ADC target gene expressions among the samples analyzed by employing differential gene expression analysis. Notably, lung adenocarcinoma tumor samples exhibited significantly increased expression of *ERBB2*, *ERBB3*, *MET*, *CEACAM5*, *CD276*, *NECTIN4*, and *PTK7*, and significantly decreased *FOLR1* expression (Fig. 1A). Despite these global differences, we identified marked inter-tumoral heterogeneity in ADC-targetable gene expression (Fig. 1B).

**Fig. 1:**
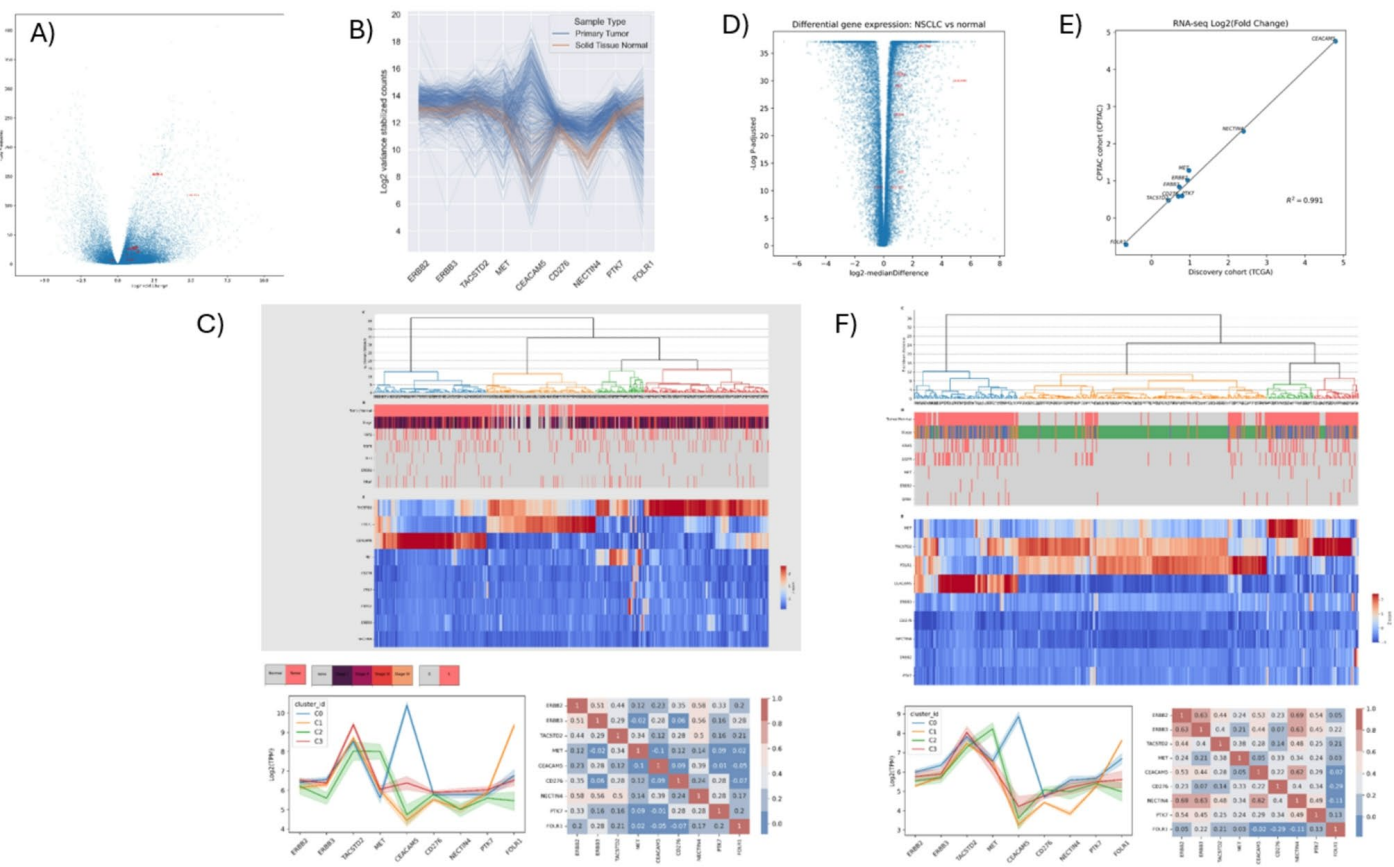
Discovery of gene expression profiles for ADC therapy in NSCLC. **(A)** Differential gene expression in NSCLC versus normal lung tissue. Log2 fold change on the X axis was plotted against negative log P-value after adjustment for false discovery rate on the Y axis. **(B)** ADC gene expression profiles across samples. Each line represents gene expression within a sample. Blue lines represent tumors samples while orange lines represent normal lung tissue samples. For each ADC target on the x axis, gene expression was plotted on the y-axis based on log2 read counts by after normalization and variance stabilizing transformation. **(C)** Hierarchical clustering of ADC-targetable gene expression. Dendrograms identify clusters of samples (columns) and genes (rows) with similar patterns of expression. Color bars beneath the top-most dendrogram correspond to cluster ID, sample type (tumor versus normal), pathologic disease stage, and driver mutational status (pink = present, gray = absent). A heatmap of ADC-targetable gene expression (rows) for each sample (columns) is colored according to increased (red) or decreased (blue) Log2 transcripts per million Z-score. **(D)** Differential gene expression in NSCLC versus normal lung tissue in the validation cohort. Log2 fold change on the X axis was plotted against negative log P-value after adjustment for false discovery rate on the Y axis. **(E)** Comparison of differential expression in ADC target genes between the discovery and validation cohorts. For each ADC target gene, the Log2 fold change in tumors versus controls was plotted in the discovery cohort (x-axis) and the validation cohort (y-axis). The correlation was assessed by Pearson’s coefficient (*r*). Linear regression was used to evaluate the coefficient of variation between both cohorts (*R*^*2*^). **(F)** Hierarchical clustering of ADC-targetable protein expression. Dendrograms identify clusters of samples (columns) and genes (rows) with similar expression patterns. Color bars beneath the top-most dendrogram correspond to cluster ID, sample type (tumor versus normal), pathologic disease stage, and driver mutational status (pink = present, gray = absent). A heatmap of ADC-targetable gene expression (rows) for each sample (columns) is colored according to increased (red) or decreased (blue) Log2 transcripts per million Z-score

This prompted us to employ unsupervised hierarchical clustering, culminating in the identification of four distinct clusters based on ADC target gene expression profiles. These clusters were categorized by: (1) a pronounced overexpression of CEACAM5 (observed in 31.5% of tumors), (2) a significant overexpression of MET (13.5% of tumors), (3) a moderate overexpression of TACSTD2 and FOLR1 (20% of tumors), and (4) a significant overexpression of TACSTD2 (34.9% of tumors). Noteworthy is that clusters characterized by high CEACAM5, MET, and TACSTD2 expressions comprised nearly all tumor samples (100%, 100%, and 99.4%, respectively. (Fig. 1C). In comparison, most normal lung tissue samples (98.3%) exhibited moderate increases in TACSTD2 and FOLR1 expressions relative to other ADC target genes. Additionally, while no driver mutations were found to be enriched across any of the identified expression clusters, BRAF mutations were significantly less common in tumors demonstrating high TACSTD2 expression (*P* = 0.93).

We examined a separate independent cohort comprising transcriptomic profiling data of 229 primary lung adenocarcinomas matched to 216 adjacent normal tissues to further validate the generalizability and robustness of ADC target gene expression results. The tumors in the validation cohort similarly exhibited significantly heightened expressions of *ERBB2*,* ERBB3*,* MET*,* CEACAM5*,* CD276*,* NECTIN4*, and *PTK7*; in conjunction with a marked decrease of *FOLR1* expression. (Fig. 1D, Supplementary Table 2). Notably, the quantitative changes in gene expression remained remarkably consistent when comparing the discovery and validation cohorts. (Figure 1E and F**)**. ADC target gene expression profiles were not associated with driver mutational status in *KRAS* (*P* = 0.50), *EGFR* (*P* = 0.15), *MET* (*P* = 0.70), *ERBB2* (*P* = 0.51), *BRAF* (*P* = 0.98). ADC target gene expression profiles were not significantly associated with the pathological disease stage (chi-squared *P* = 0.055).

To characterize differential protein expression in NSCLC, we analyzed 110 tumors and 101 matched normal tissues from the validation cohort using RNA-seq and tandem mass tag (TMT-10) mass spectrometry. Tumors exhibited significantly elevated expression of HER2, MET, CEACAM5, B7-H3, NECTIN4, and CCK4, while normal lung tissue showed higher levels of HER3, Trop-2, and FRα (Fig. 2A; Supplementary Table 3). Protein and gene expression were moderately to strongly correlated (Pearson *r* = 0.69; R² = 0.66; Fig. 2B). Strong positive correlations (Pearson’s *r* > 0.7) were observed for HER2, MET, CEACAM5, NECTIN4, and FRα, with predictive models achieving R² = 0.61–0.82 (Fig. 2C and D). RNA-seq and mass spectrometry results were concordant for most ADC targets, except ERBB3/HER3 (Supplementary Tables 2 & 3). Although the RNA-protein correlation for TACSTD2 was moderate (Pearson *r* = 0.5, R² = 0.26), distinct overexpression patterns at both transcriptomic and proteomic levels were observed in a subset of NSCLC tumors. This suggests that TACSTD2 may remain a viable ADC target in selected patients, particularly when protein expression is confirmed. These findings underscore the importance of integrating multi-omic data in ADC biomarker development to guide therapies.

**Fig. 2:**
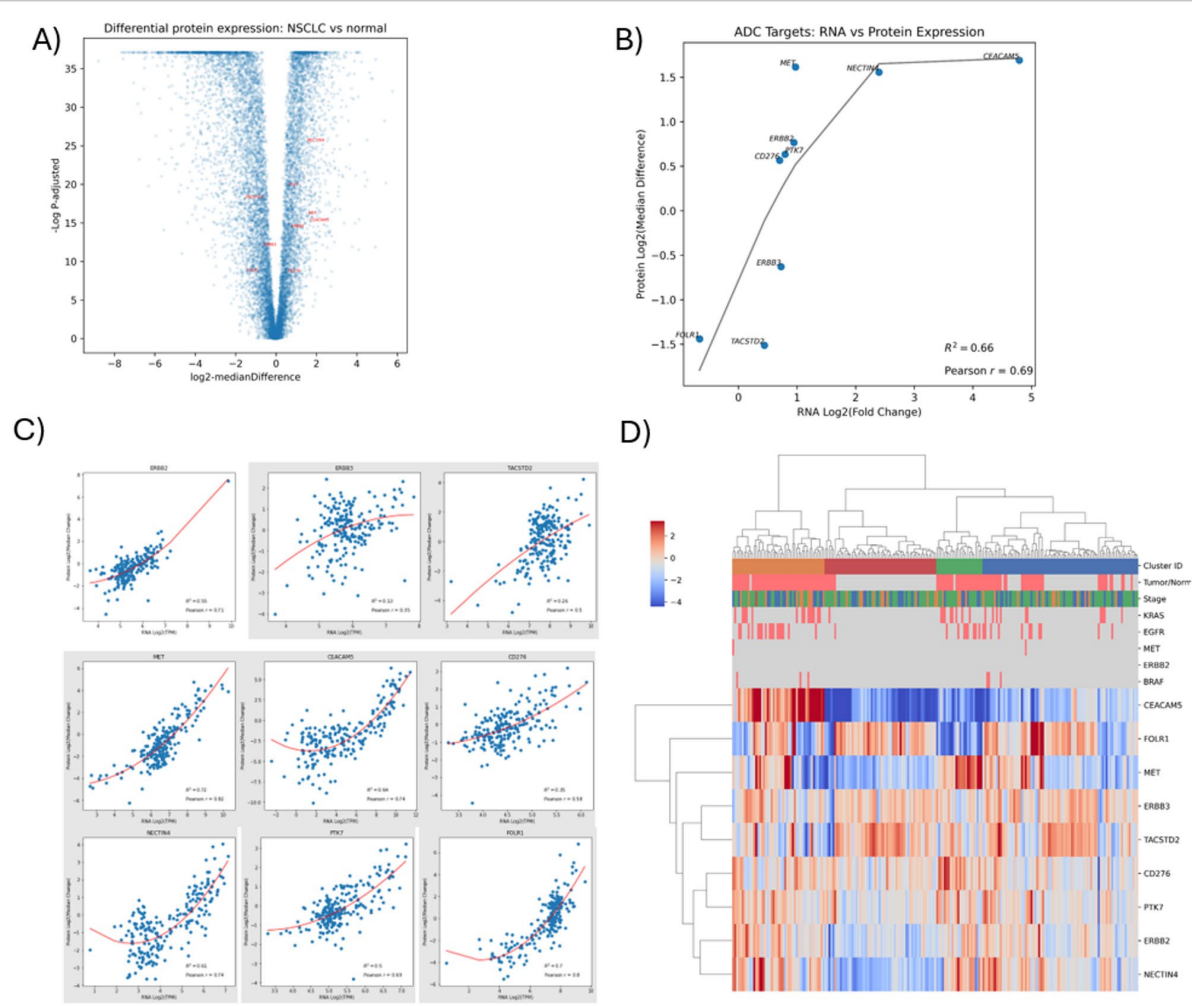
Proteomic profiles for ADC therapy in NSCLC. **(A)** Differential protein expression in the validation cohort. A volcano plot shows Log2 median differences in protein expression between tumors versus paired normal tissue were plotted against negative log-transformed P-values after adjustment for false discovery rate < 0.01 using the Benjamini-Hochberg correction. **(B)** Comparison of differential ADC target gene and protein expression. Differential protein expression measured by Log2 median difference in tumors versus matched controls (y-axis) was compared to differential gene expression measured by Log2 fold change by RNA-Seq (x-axis). The correlation was assessed by Pearson’s coefficient (*r*). Second-degree polynomial regression was used to determine the coefficient of variation between protein and gene expression (*R*^*2*^). **(C)** Gene versus protein expression for each ADC target. The Pearson correlation coefficient (*r*), second-degree polynomial regression model (red line), and coefficient of variation (*R*^*2*^) for protein versus gene expression for each ADC target. **(D)** Hierarchical clustering of ADC-targetable protein expression in the validation cohort. Dendrograms identify clusters of samples (columns) and genes (rows) with similar patterns of expression. Color bars beneath the top-most dendrogram correspond to cluster ID, sample type (tumor versus normal), pathologic disease stage, and driver mutational status (pink = present, gray = absent). A heatmap of ADC-targetable protein expression (rows) for each sample (columns) is colored according to increased (red) or decreased (blue) Log2 median difference in protein expression

In conclusion, this study highlights distinct, mutually exclusive proteogenomic patterns of ADC target expression in NSCLC, providing insights into the tumor’s molecular heterogeneity. These findings offer a framework for stratifying patients based on unique proteogenomic profiles, enabling the potential prediction of ADC therapy response and paving the way for personalized treatment approaches. Furthermore, strong RNA-protein correlations support the additional value of transcriptomic biomarkers as a complementary approach to immunohistochemistry for identifying ADC-eligible patients, especially in settings where tissue availability is limited. Although the targets of ADCs are typically membrane-bound proteins, their expression may not be fully captured by RNA sequencing assays. Nevertheless, our findings suggest that distinct NSCLC subtypes, characterized by differential ADC target expression, may inform a more precise selection of ADC therapies, such as CEACAM5-directed (e.g., tusamitamab ravtansine), MET-directed (e.g., telisotuzumab vedotin), and TACSTD2-directed (e.g., datopotamab deruxtecan) treatments, thereby optimizing patient stratification and therapeutic efficacy. By moving beyond a one-size-fits-all strategy, this tailored approach could transform ADC utilization in NSCLC. However, further validation through randomized clinical trials is essential to confirm the clinical utility of these proteogenomic patterns as predictive biomarkers.

## Electronic supplementary material

Below is the link to the electronic supplementary material.

Supplementary Material 1

## Data Availability

No datasets were generated or analysed during the current study.
