# Supplementary material for: Advancing precision antibody-drug conjugate therapy: unique proteogenomic profiles of tumor subsets in non-small cell lung cancer
Source: Exp Hematol Oncol. 2025 Jul 11;14:96. doi: 10.1186/s40164-025-00685-w (PMC12247227; doi:10.1186/s40164-025-00685-w)
Supplement: Supplementary file 1 — Supplementary Material 1 [file 40164_2025_685_MOESM1_ESM.docx]

**Methods:** A systematic search of the Medline (Ovid), Embase (Elsevier), and CENTRAL (Cochrane Library) databases, as well as peer-reviewed literature in the English language, conference abstracts, and trial registrations from major international oncology meetings up to March 2023, were conducted. The search was restricted to studies written in or translated into English and discussed keywords such as “Antibody Drug Conjugates”, “non-small cell lung cancer,” “NSCLC,” “ADC,” and “lung adenocarcinoma.” Clinical trials describing ADC-based interventions in NSCLC were identified by reviewing each title and abstract. Targets of interest were then identified based on this information.

A total of 5,676 references were identified through the electronic search; 1,847 potentially relevant articles remained for screening after removing duplicated studies, and the search was restricted to adult humans and published in English. After applying the selection criteria, 35 studies remained for full assessment, from which we extracted data on the target antigens for different ADCs. ADC targets included TROP2, MET, CEACAM5, HER2, HER3, Nectin4, PTK7, FRα, B7H3. Drug targets, target genes, and corresponding ADCs are summarized in **Table 1**.

Table 1: ADC Targets of Interest in Non-Small Cell Lung Cancer

| Drug Target | Gene ID | Drug Name |
| --- | --- | --- |
| TROP2 | *TACSTD2* | Sacituzumab govitecan (IMMU-132) |
| MET | *MET* | Telisotuzumab vedotin (ABBV-399) |
| CEACAM5 | *CEACAM5* | SAR408701 |
| HER2 | *ERBB2* | Trastuzumab deruxtecan (DS-8201a) |
| HER3 | *ERBB3* | Patritumab deruxtecan (U3-1402) |
| Nectin 4 | *NECTIN4* | Enfortumab vedotin (ASG-22CE) |
| PTK7 | *PTK7* | PF-06647020 |
| FRα | *FOLR1* | IMGN853 (Mirvetuximab soravtansine) |
| B7H3 | *CD276* | MGC018 |

*Discovery cohort*

We downloaded RNA-sequencing data from the National Cancer Institute Genomic Data Commons (<https://gdc.cancer.gov/>) to charactƒerize ADC target gene expression from project TCGA-LUAD. The experimental design and methods for next-generation sequencing are detailed elsewhere. We obtained RNA-seq read count files and somatic variant data from 537 primary lung adenocarcinoma (LUAD) and 59 normal lung tissue samples, which are tumor-adjacent samples from the TCGA.

*Validation cohort:*

To validate the clusters identified in the discovery cohort, we performed similar analyses in a separate independent cohort of patients from the CPTAC lung adenocarcinoma project. Genomic and transcriptomic data files for this project were downloaded from the Genomic Data Commons (GDC) (<https://portal.gdc.cancer.gov/>) via dbGaP Study Accession number phs001287.v5.p4. Proteomics datasets were obtained through the CPTAC data portal (<https://cptac-data-portal.georgetown.edU/cptac/s/S056>). Data used in this publication were generated by the National Cancer Institute Clinical Proteomic Tumor Analysis Consortium.

*Bioinformatics and statistics*:

The GDC mRNA quantification analysis pipeline was used to create RNA-seq read count files, wherein STAR[7] was used for read alignment followed by quantification of raw read counts and calculation of Fragments per Kilobase of transcript per Million mapped reads (FPKM), upper quartile normalized FPKM (FPKM-UQ), and Transcripts per Million (TPM). Pre-alignment and post-alignment quality assessments were performed with FASTQC and Picard Tools, respectively. The GDC DNA-seq analysis pipeline was used for somatic variant calling. The GDC Variant aggregation pipeline generates Mutation Annotation Format (MAF) files containing only somatic variants. MAF files were used for downstream mutational analyses.

The DESeq2 software package was utilized to perform differential gene expression analysis. Criteria for significant gene expression included a mean read count > 5, Log2 Fold Change greater than 1, and false discovery rate-adjusted P-value < 0.05. Within-sample ADC target gene expression comparisons were compared using transcripts per million (TPM). Hierarchical clustering was used to identify groups of tumors with similar intratumoral profiles of ADC-targetable gene expression. The Ward variance minimization algorithm was used to determine distances between sample gene expression profiles. Chi-squared residual analysis was used to determine associations between gene expression clusters and discrete variables (i.e. disease stage, driver mutations). Pearson’s *r* was used to assess the correlation between RNA and protein expression*.* Generalized linear models were fitted to protein and gene expression to determine the agreement between these two molecular variables.

**Supplementary Table 1:** Differential gene expression of ADC targets in the discovery cohort

| **Gene** | **Log2 Fold Change** | **pvalue** | **FDR-adjusted P-value** |
| --- | --- | --- | --- |
| **ERBB2** | 1.01259 | 1.81E-13 | 1.17E-12 |
| **ERBB3** | 0.833992 | 1.8E-12 | 1.06E-11 |
| **TACSTD2** | 0.472822 | 0.000962 | 0.002071 |
| **MET** | 1.277953 | 8.06E-10 | 3.72E-09 |
| **CEACAM5** | 4.759473 | 3.72E-53 | 2.6E-51 |
| **CD276** | 0.581761 | 7.21E-12 | 4.04E-11 |
| **NECTIN4** | 2.335266 | 6.63E-69 | 9.13E-67 |
| **PTK7** | 0.593573 | 1.49E-07 | 5.38E-07 |
| **FOLR1** | -0.72017 | 0.002497 | 0.005016 |

**Supplementary Table 2:** Differential gene expression of ADC targets in the validation cohort

| **Gene** | **Log2 Fold Change** | **P-value** | **FDR-adjusted P-value** |
| --- | --- | --- | --- |
| **ERBB2** | 0.9465 | 8.88E-15 | 3.81E-14 |
| **ERBB3** | 0.7278 | 1.89E-11 | 5.28E-11 |
| **TACSTD2** | 0.4434 | 1.72E-05 | 2.88E-05 |
| **MET** | 0.9747 | 1.02E-06 | 1.87E-06 |
| **CEACAM5** | 4.7922 | 2.77E-14 | 1.1E-13 |
| **CD276** | 0.7026 | 6.8E-15 | 2.98E-14 |
| **NECTIN4** | 2.3979 | 2.33E-17 | 2.09E-16 |
| **PTK7** | 0.7964 | 8.24E-14 | 3.07E-13 |
| **FOLR1** | -0.6631 | 1.72E-05 | 2.88E-05 |

**Supplementary Table 3:** Differential protein expression of ADC targets in the validation cohort

| **Gene** | **Log2 Median Difference** | **P-value** | **FDR-adjusted P-value** |
| --- | --- | --- | --- |
| **ERBB2** | 0.7653 | 2.81E-07 | 5.38E-07 |
| **ERBB3** | -0.6283 | 3.35E-06 | 5.88E-06 |
| **TACSTD2** | -1.5129 | 5.65E-09 | 1.26E-08 |
| **MET** | 1.6138 | 4.92E-08 | 1.01E-07 |
| **CEACAM5** | 1.6912 | 1.24E-07 | 2.45E-07 |
| **CD276** | 0.566 | 0.000117 | 0.000181 |
| **NECTIN4** | 1.5566 | 2.52E-12 | 7.84E-12 |
| **PTK7** | 0.6335 | 1.08E-09 | 2.57E-09 |
| **FOLR1** | -1.4403 | 0.000106 | 0.000165 |
